# Molecular breeding of water lily: engineering cold stress tolerance into tropical water lily

**DOI:** 10.1038/s41438-018-0086-2

**Published:** 2018-11-30

**Authors:** Cuiwei Yu, Guirong Qiao, Wenmin Qiu, Dongbei Yu, Shirong Zhou, Yan Shen, Guanchun Yu, Jing Jiang, Xiaojiao Han, Mingying Liu, Liangsheng Zhang, Fei Chen, Yuchu Chen, Renying Zhuo

**Affiliations:** 1 State Key Laboratory of Tree Genetics and Breeding, Chinese Academy of Forestry, 100091 Beijing, China; 2 Zhejiang Humanities Landscape Co., Ltd., Hangzhou Tianjing Aquatic Botanical Garden, 310000 Hangzhou, Zhejiang China; 3 Key Laboratory of Tree Breeding of Zhejiang Province, The Research Institute of Subtropical forestry, Chinese Academy of Forestry, 311400 Hangzhou, Zhejiang China; 4 State Key Laboratory of Ecological Pest Control for Fujian and Taiwan Crops, Key Laboratory of Ministry of Education for Genetics, Breeding and Multiple Utilization of Crops, Fujian Provincial Key Laboratory of Haixia Applied Plant Systems Biology, Fujian Agriculture and Forestry University, 350002 Fuzhou, China

## Abstract

Water lilies (order Nymphaeales) are rich in both economic and cultural values. They grow into aquatic herbs, and are divided into two ecological types: tropical and hardy. Although tropical water lilies have more ornamental and medicinal values compared to the hardy water lily, the study and utilization of tropical water lilies in both landscaping and pharmaceutical use is greatly hindered due to their limited planting area. Tropical water lilies cannot survive the winter in areas beyond 24.3°N latitude. Here, the transgenic pipeline through the pollen-tube pathway was generated for water lily for the first time. To improve cold stress tolerance of tropical water lilies, a gene encoding choline oxidase (*CodA*) driven by a cold stress-inducible promoter was transformed into a tropical water lily through the pollen-tube transformation. Six independent transgenic lines were tested for survival rate during two winter seasons from 2015 to 2017 in Hangzhou (30.3°N latitude). PCR and southern blot detection revealed that the *CodA* gene had been integrated into the genome. Reverse transcription PCR showed that *CodA* gene was induced after cold stress treatment, and further quantitative real-time PCR revealed different expressions among six 4 lines and line 3 had the highest expression. Multiple physiological experiments showed that after cold stress treatment, both the conductivity and malondialdehyde (MDA) levels from transgenic plants were significantly lower than those of non-transgenic plants, whereas the content of betaine and the activity of superoxide dismutase, catalase, and peroxidase were higher than those from non-transgenic plants. These results suggest that expression of exogenous *CodA* gene significantly improved the cold stress tolerance of tropical water lilies through a wide range of physiological alterations. Our results currently expanded a six-latitude cultivating area of the tropical water lilies. These results not only illuminate the bright future for water lily breeding but will also facilitate the functional genomic studies.

## Genetics: The crop that came in from the cold

Pioneering work in the genetic modification of water lilies lays the foundation for engineering more robust strains of this agriculturally valuable crop. Tropical water lilies are used to manufacture products including tea, food and essential oils, but their cultivation is limited by sensitivity to cold. Researchers led by Fei Chen at Fujian Agriculture and Forestry University have developed a genetic engineering procedure for introducing favorable new traits into these plants, offering an efficient alternative to labor-intensive crossbreeding procedures. As an initial demonstration, the researchers engineered tropical water lilies with a gene that confers enhanced cold tolerance, allowing the plants to survive winter temperatures that would otherwise prove inhospitable. This process should accelerate development of lilies that grow in a more diverse range of climates, and enable more extensive genomic analysis of this crop.

## Introduction

Water lily is a common name for all plants categorized into the order Nymphaeales. Most of water lilies (more than 50 species) are categorized within the ornamental-enriched genus *Nymphaea*^1^. Many *Nymphaea* species are world famous and important aquatic plants^2^ with a wide range of colors, long blooming periods, high stress tolerance, and adaptability. In addition, water lilies have been used in landscaping^3^, sewage treatment^4–6^, decoration^7^, floral arrangements, cuisine, beverage^8^, cosmetic products, essential oils, and medicine^9–11^. The *Nymphaea* water lily is divided into two ecological types:^12^ tropical water lily (includes *Brachyceras*, *Anecphya*, *Lotos*, and *Hydrocallis*) and hardy water lily (*Nymphaea* spp.). The tropical water lilies generally have higher ornamental and economic values than the hardy water lilies do. The flowers of tropical water lily are rich in color, with red, yellow, white, blue, purple, and other complex color constitutions. Additionally, leaves of the tropical water lily are in reddish, purplish, or green color and vary in shape. Some leaves even have red spots. Also, the leaf margin of the water lily has regular or irregular serrated pattern. Tropical water lilies are very popular in tropical countries, for example, *Nymphaea stellata* and *Nymphaea nouchali* are the national flower in Sri Lanka and Bangladesh, respectively. In contrast, hardy water lilies only exhibit three petal colors: red, white, and yellow. Another phenotypic distinction between hardy and tropical water lilies is the lack of spots and serration in leaves of hardy water lily. The flower of the *Brachyceras* tropical water lilies have fragrance, which makes it useful for making flower tea. Additionally, the pedicels and petioles of *Lotos* tropical water lily are edible and have been widely consumed in Thailand as a vegetable. Tropical water lilies have also been utilized in bouquets, essential oils, and wines. Unfortunately, the promotion of the tropical water lily is seriously limited by its inability to overwinter in north subtropical (24.3°N latitude) or more northern areas. Therefore, breeders have strong interest to expand the water lily market, by improving the cold stress tolerance of the tropical water lily so that it can overwinter in the cold areas.

Currently, crossbreeding is a major technique for breeding novel water lilies. This is partly due to the unavailability of the water lily genome and the limited molecular information. Most of the cultivated garden water lilies are hybrid varieties selected by breeders, while others are natural mutants. The history of artificial crossbreeding of water lilies can be traced back to 166 years ago by the British breeder Joseph Paxton in 1851^13^, who crossed two species from *Lotos* subgenus and obtained the first hybrid, *Nymphaea* “Devonishire.” The water lily breeding history in China started roughly at the end of the twentieth century when Mr. Guozhen Huang (from Wuhan Botanical Garden, Chinese Academy of Sciences) began to crossbreed water lilies. In recent years, multiple water lily breeding organizations have emerged in China, such as Xi’an Botanical Garden, Nanjing Yileen Garden, Hainan Lianhua Ecological Culture Development Co., Ltd., and Zhejiang Humanities Landscape Co., Ltd.^14^

The water lily breeders have long attempted to crossbreed the tropical and hardy water lilies with the idea that such hybrid would display the advantages of both parents. Currently, the value of tropical water lily is negatively affected by its inability to overwinter in regions northern than the 24.3°N latitude, or the north subtropical regions. Thus, it is a major challenge for worldwide water lily breeders to improve the cold stress tolerance of the tropical water lily. Since the French breeder Joseph B. Latour-Marliac tried to hybridize tropical water lilies and hardy water lilies, water lily breeding for cold stress tolerance has been attempted for more than 150 years^13^. Although inter-subgeneric hybrids of the “Siam” series varieties were later developed by Thai breeders, these hybrids exhibit only a single color and lack the fragrant traits from tropical water lily^15^. Hence, the goal remains to breed a water lily with characteristics of underwater part of the hardy water lily and above water portion retaining the characteristics of the tropical water lily. Currently, using traditional hybrid techniques to improve the cold resistance is not feasible. In the past few years, with the development of plant molecular biotechnology, a lot of achievements have been made in the study of improving plant resistance to cold stress.^16–18^ Pollen-tube pathway transgenic technology refers to injecting a DNA fragment that harbors the target gene into the pollen-tube channel by pollination^19,20^. The target gene, such as the cold stress-tolerant gene, could be carried by the pollen tube into the embryonic sac and then goes into early embryonic cells or the zygotes. This method does not need the cell or tissue culture system, enabling the direct and easy acquisition of the cold stress-tolerant transgenic seeds. Currently, as far as we know, although pollen-tube pathway has been successfully applied in multiple crops for gene functional analyses and molecular breeding, it has not been applied in the water lilies.

In this study, we generated the first transgenic pipeline for the water lilies using pollen-tube pathway. The cold stress-tolerant choline oxidase (*CodA*) gene was successfully transformed into the tropical water lily using multiple exams, and induced by cold stress both in the lab and in the field test. This transgene activated a series of physiological responses to combat cold. Field tests showed that a transgenic tropical water lily exhibits a strong cold stress tolerance that could survive in Hangzhou city, China, expanding the cultivation area by six latitudes. Our results lay the foundation for molecular breeding of water lilies, and also illuminate the future of functional genomics in water lily research.

## Materials and methods

### Water lily flower and pollen collection

**Fig. 1:**
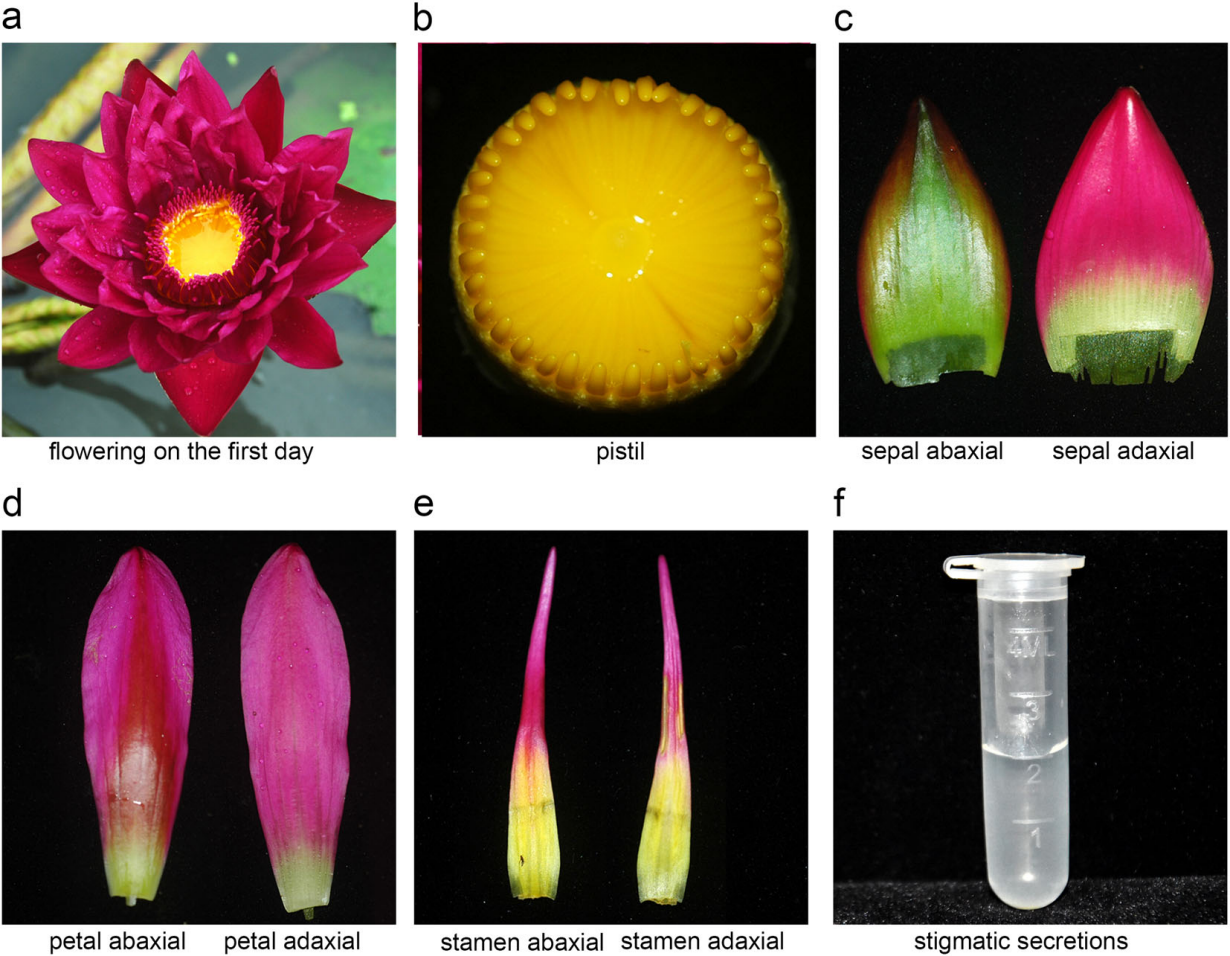
Flower organs of a tropical water lily. **a** Flowering on the first day. **b** Pistil. **c** Sepals (abaxial and adaxial view). **d** Petals (abaxial and adaxial view). **e** Stamens (abaxial and adaxial view). **f** Stigmatic secretions in a centrifuge tube

The blooming period of the tropical water lily lasts from June to early November in Hangzhou, China. Each single flower has a blooming period of 3–5 days. The transformation efficiency of pollen-tube pathway is usually low as compared to the tissue culture-based transformation^21^, so this study needs a large number of flowers. The flower is dichogamous on the first day of flowering, that is, when the pistils are ripe but the stamens are not ready yet (Fig. 1a). There are abundant stigmatic secretions on the stigma disk on the first day of flowering, but become only a few from the second day of flowering (Fig. 1b–f). The stamens ripen from outside whorl to inside whorl. The flowers that opened on the first day were picked off and grown in a bottle with a half bottle of water. These bottles were then placed in a 25 °C incubator in order to avoid insect pollination. The stamens were cut off on the third day of flowering and were placed into a sterile sealed bottle in reserve.

### Gene, plasmid, and *Agrobacterium*

**Fig. 2:**
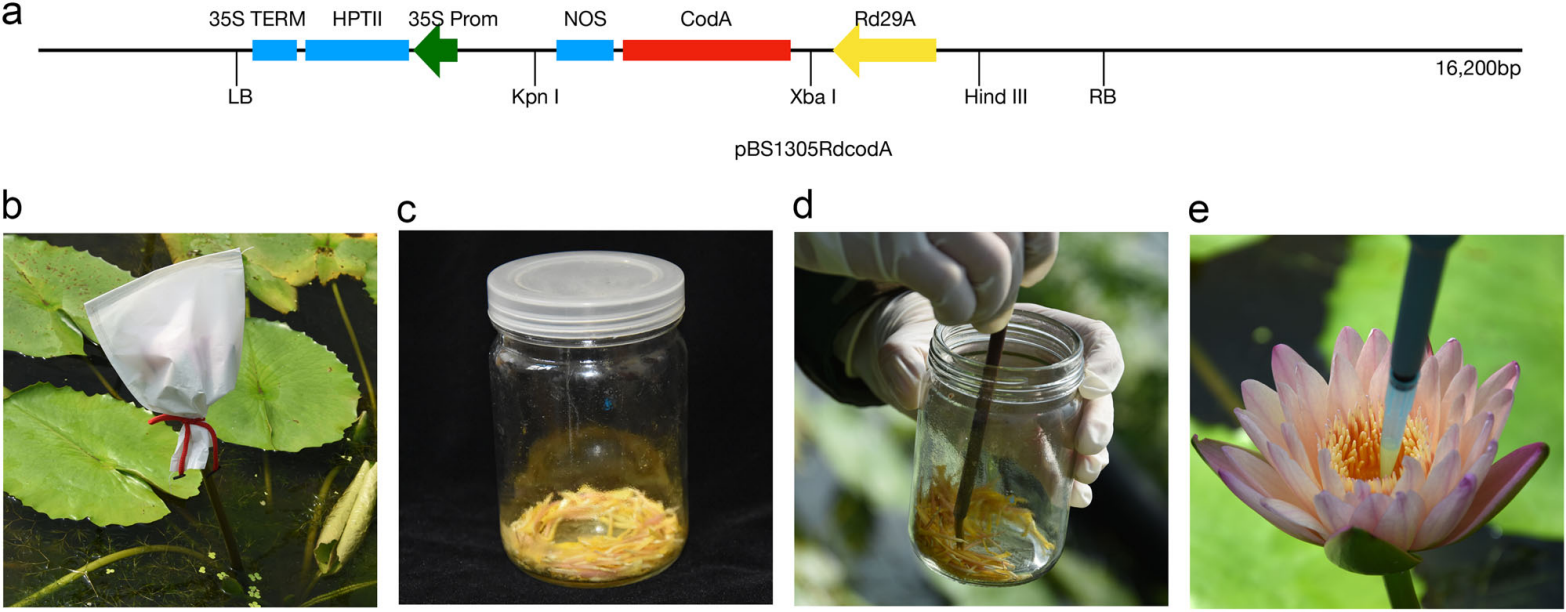
Key steps of the water lily gene transformation procedure. **a** The plasmid used for transformation. **b** Pollinated flower with a paper bag. **c** Collected stamen and pollen. **d** Collecting pollen with a brush. **e** Pour the mixture onto the stigma

In this research, the *CodA* gene was selected for gene transformation. This gene is encoded by soil bacteria that synthesize betaine by oxidizing choline without cofactors^22^. Because the choline dehydrogenase and choline oxidase can be combined with the synthesis of glycine betaine, the betaine synthase gene is considered efficient and one of the most promising stress resistance genes^23^. The *CodA* gene is 2400 bp in length, with a 1641-bp open-reading frame encoding 547 amino acid residues^24^. *CodA* gene has been successfully applied to have improved plant resistance in both eudicot *Arabidopsis* and monocot rice^25–30^. The promoter *Rd29A*, activated by drought, salinity, and chilling stresses, was employed to induce the *CodA* expression on plasmid pBS1305RdcodA^31^ (Fig. 2a). The *Agrobacterium* strain, EHA105, was inoculated into yeast-mold medium^32^ supplemented with 50 mg/L kanamycin and 50 mg/L rifampicin, grown in streak, with room temperature 28 °C for 3 days. A tropical hybrid individual of *Nymphaea* sp. with lots of pollen was chosen from Zhejiang Humanities Landscape Co., Ltd. A tropical hybrid individual of *Nymphaea* sp. with high setting rate was chosen for the female parent.

### Plant transformation procedure

The afternoon before the day of pollination is the best time for female parent preparation. Flowering buds that would flower on the next day (the buds were upright and sepals were slightly loose) were bagged with paper bags (Fig. 2b). At the first flowering day, the paper bag on the flower was removed. The stigmatic secretion of the flower was put into a 5-mL PE tube using a pipette, and then *Agrobacterium* was gently scraped into the tube with a small plastic spoon. The *Agrobacterium* was suspended to OD600 = 0.7 with the stigmatic secretion. The pollen was collected (Fig. 2c) and put into the same tube with a brush. The *Agrobacterium*, pollen, and stigma secretion were mixed evenly in the PE tube using a pipette (Fig. 2d). Everything was then immediately put into the stigma using a pipette (Fig. 2e). PE tubes, spoons, brushes, and pipette tips were used only once each time. After pollination, a label with all the information was hung on pedicels, and the flower was bagged with the same bag. Three days after pollination, the bag was removed.

### Seed treatment

After a single flowering period, flowers went back into the water to develop fruit. Since the seed size of the tropical water lily is very small, it is easily affected by a variety of environmental and ecological factors after germination. These factors include temperature and light, *Margarya*, *Spirogyra*, tadpoles, and competitive aquatic plants. The water lily germination rate is relatively low. Considering these factors, we planted hundreds of seeds from each parent in order to ensure the appropriate number of seedlings. About 1 month after pollination, the seeds were mature and were collected and selected for germination in water with 10 mg/L hygromycin B (HygB). After the submerged leaves and new root emerged, the seedlings were transplanted to the soil.

### Overwinter test

The seedlings were transplanted into the seedling cups when five leaves emerged. They were transplanted into a plastic bowl of 18 cm in diameter once they had 2–3 floating leaves in the seedling cups. Once the seedlings had 5–6 floating leaves (about 10 cm in diameter), they were transplanted into the outdoor field. In the late autumn (early November in Hangzhou), the number of plants was counted in the open field. Water level was kept ~20 cm above the bottom in the winter. In the coming spring (late April of Hangzhou), the number of plants with floating leaves was counted. The growth and development of living plants were observed continuously.

### PCR detection

Six putatively transformed plants that grew well in winter and a non-transgenic plant were used when they displayed the fingernail-sized leaves. The cetyltrimethylammonium bromide method^33^ was used to extract genomic DNA. DNA quality and concentrations were evaluated using agarose gel electrophoresis and a NanoDrop-2000 (Thermo Fisher). Three pairs of primers were designed to amplify the *Rd29A* promoter, *HYG* gene, and *CodA* gene by PCR (Table 1). The PCR products were resolved in 0.8% (w/v) agarose gels and stained with DNA Green (Tiandz Inc., Beijing, China).

**Table 1:** The PCR primers used in this study

Gene name	Primer	Primer sequence (5′–3′)
*Rd29A*	*RD29A*-Forward	GACTCAAAACAAACTTAC
	*RD29A*-Reverse	CAAACCCTTTATTCCTG
*Hyg*	*Hyg*-Forward	GTTTATCGGCACTTTGCATCG
	*Hyg*-Reverse	GGAGCATACGCCCGGAGT
*CodA*	*CodA*-Forward	ATCGGGCTACGACTGGGACT
	*CodA*-Reverse	CAACAGCTTCGGCGTATCG
*Actin*	*Actin*-Forward	ATGGAACTGGAATGGTCAAG
	*Actin*-Reverse	TCAAGCTCTTGCTCATAGTC
*CodA* (qRT-PCR)	*CodA*-Forward	AATCGGGCTACGACTGG
	*CodA*-Reverse	GCGTCCTCGTTGGTTTC
*Actin* (qRT-PCR)	*Actin*-Forward	TGTGGCCATTCAAGCTGTTC
	*Actin*-Reverse	ACCAGCAAGATCAAGACGGA

### Reverse transcription PCR

To detect whether the transgene is responsive to cold stress, we selected a stress-inducible promoter *Rd29A* to induce *CodA* gene expression. Two plants with best growth and multiple ramets were selected and grown at 25 °C (water temperature) for 1 month, and then the plants were moved from 25 °C to 4 °C (water temperature) for 24 h to induce cold response. Leaves were harvested from the plants before and after the cold treatment. Total RNA was isolated using the RNAprep Pure Plant Kit (polysaccharides and polyphenolics-rich) (TIANGEN, Beijing, China). First-strand complementary DNA (cDNA) synthesis was performed using SuperScript III First-Strand Synthesis System for reverse transcription PCR (RT-PCR) (Invitrogen, Carlsbad, CA, USA). The *Actin* gene^34^ of water lily was used as an endogenous control. The *Actin* and the *CodA* gene primers are shown in Table 1. The RT-PCR products were analyzed using 0.8% (w/v) agarose electrophoresis.

### Quantitative real-time PCR

RNA extraction and cDNA synthesis was performed as mentioned above. Quantitative real-time PCR (qRT-PCR) was performed on a 7300 Real-Time PCR System (Applied Biosystems, Foster City, CA, USA) using a SYBR PrimeScript^TM^ RT-PCR Kit (TaKaRa, Dalian, China). Relative gene expression was estimated based on the 2^−ΔΔCT^ method^35^. The primers of the housekeeping gene (*actin*) and the *CodA* gene for qRT-PCR are listed in Table 1. Each line was repeated with three biological repeats.

### Southern blot

Southern blotting was performed using a DIG-High Prime DNA Labeling and Detection Starter Kit II (Roche, Mannheim, Germany) according to the manufacturer’s instructions. Six putative transgenic plants (>50 cm in height) and one control plant were selected. Genomic DNA (30 μg/sample) was digested with *Hin*dIII three times (2 h, 16 h, and 4 h) at 37 °C. The digested DNA samples were separated using 1% (w/v) agarose gel electrophoresis and transferred to a Hybond N+ nylon membrane. The *Hyg* gene probe was prepared using a PCR DIG Probe Synthesis Kit (Roche). Hybridization, washing, and signal detection were performed according to the manufacturer’s instructions.

### Transformation efficiency

After the fruits were ripened, the transformed seeds were collected, and from these 1000 seeds were randomly chosen. Three biological replicates for this step were selected. The seeds were placed in water containing 10 mg/L Hyg for screening. The well-developed seedlings were selected and moved to the soil. When the seedlings had two to three floating leaves, the transgene was detected using PCR. The transformation rate = transformed seedlings/1000 seeds. The average number of the transformation rate was considered.

### Cold treatment and physiological test

Six positive transgenic lines and one control were used for cold stress analysis. Each line was divided into three clones and was grown in a greenhouse for 1 month with water temperature of 25 °C. After 1 month, these plants were moved to 4 °C (water temperature) for 24 h. Conductivity^36,37^ was measured in electrolyte leakage rate to show the damage level of the cell membrane. In the cold stress, the cell membrane is destroyed and the conductivity (measured by electrolytic leakage rate) is larger, indicating that the cell membrane is destroyed severely. Fresh leaf tissue (0.2 g) was harvested from each plant and put into a test tube with 5 ml of deionized water, oscillated for 1.5–2 h at 25 °C, and then conductivity was measured using a conductivity meter. The tube was put into boiling water for 30 min, and then cooled to 25 °C. Next, electrolytic leakage rate was determined using this formula: electrolytic leakage rate (%) = conductivity value/conductivity value after boiling × 100%. Superoxide dismutase (SOD) activity^38^, MDA content^39^, catalase (CAT) activity^40^, peroxidase (POD) activity^41^, and GB content^42^ were measured by a spectrophotometer using a series of kits (COMIN, Suzhou, China).

### Statistical analysis

All analyses were set up with three biological replicates. Data analyses were conducted using SPSS version 18.0 (SPSS Inc., Chicago, IL, USA).

## Results

### The established transgenic system of tropical water lily

The complete transgenic system of pollen-tube pathway was developed for tropical water lily as illustrated in Fig. 2, which presents the plasmid and the whole transformation procedures. The *Agrobacterium* suspension, carrying plasmid pBS1305RdcodA (integrated with *CodA* gene and 35S promoter), was mixed with the stigmatic secretions. We compared the OD600 value of the mix at 0.47, 0.93, and 1.05 (Table S1) for best infectious rate. And finally we found the most suitable mix had OD600 = 0.7, which has the highest transformation rate and setting rate of mature plants. We used the mix to infect the flower on the first day of flowering. Seeds were selected for germination in water with HygB. Ten micrograms per liter was the best concentration of HygB for seed selection after a series of tests with HygB concentrations: 0, 5, 10, 15, 20, and 25 mg/L (Table S2).

### The putatively transformed plants successfully overwintered outside in Hangzhou (30.3°N)

**Fig. 3:**
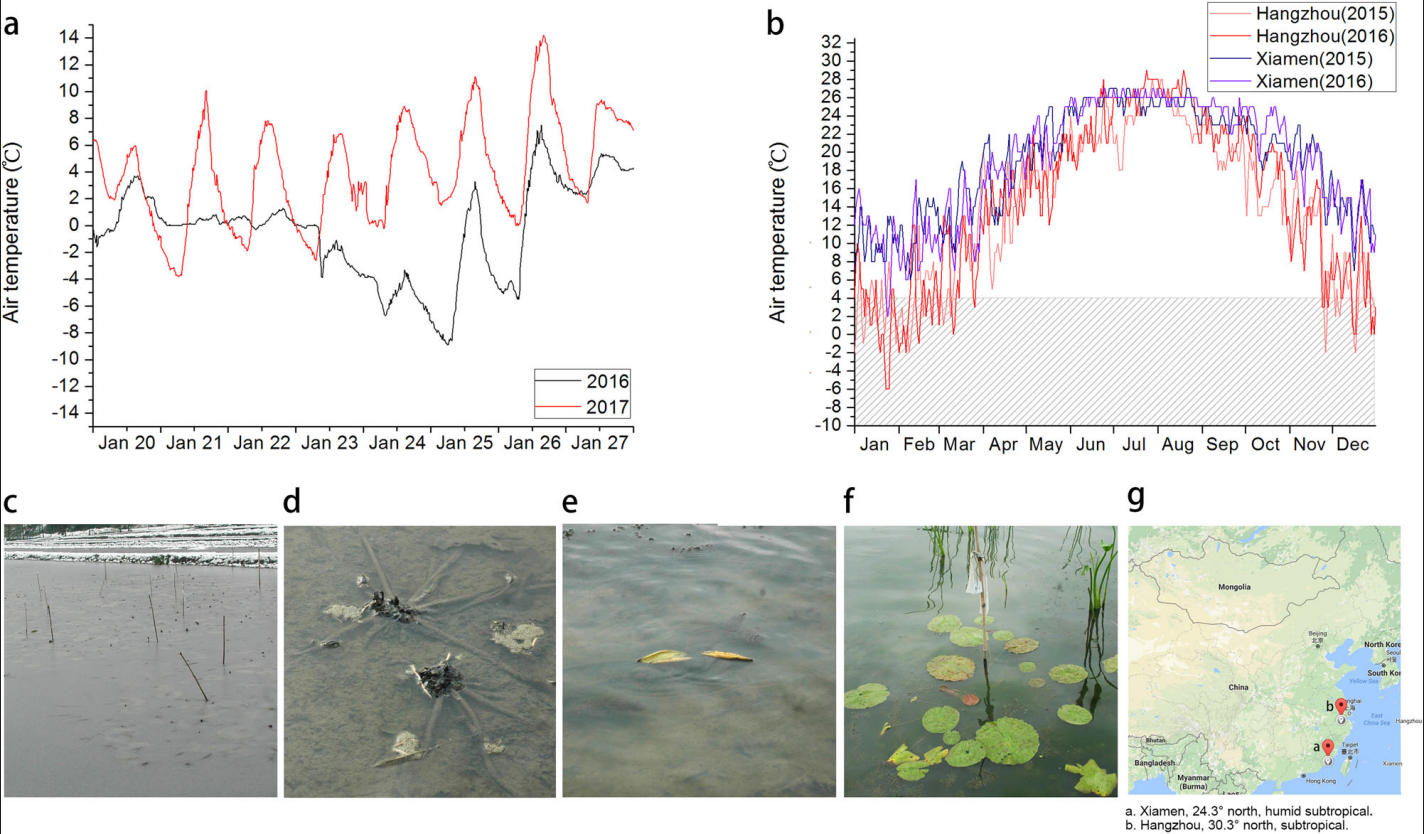
Overwinter test of transformed water lilies. **a** The air temperature between 20 and 28 Janurary, the coldest days in winter of 2015–2016 and 2016–2017 measured by the agricultural meteorology monitor (TOP, Zhejiang, China) in the water lily field. **b** Comparison of the daily minimum air temperature in 2015 and 2016 of Hangzhou and Xiamen. Xiamen is the far north location that tropical water lilies could overwinter with almost the whole year above 4 °C. **c** Hangzhou, December 6, 2015, water surface of the whole field was frozen. **d** The non-transgenic control water lily died in freezing cold water. **e** The two fresh leaves in the ice on February 15, 2016 (the surface of the water was frozen, but under the ice the water was flowing). **f** The floating leaves on May 5, 2016. **g** Our cold stress-tolerant water lily moved the growth-limitation latitude from **a** Xiamen (24.3°N latitude) to **b** Hangzhou (30.3°N latitude)

**Fig. 4:**
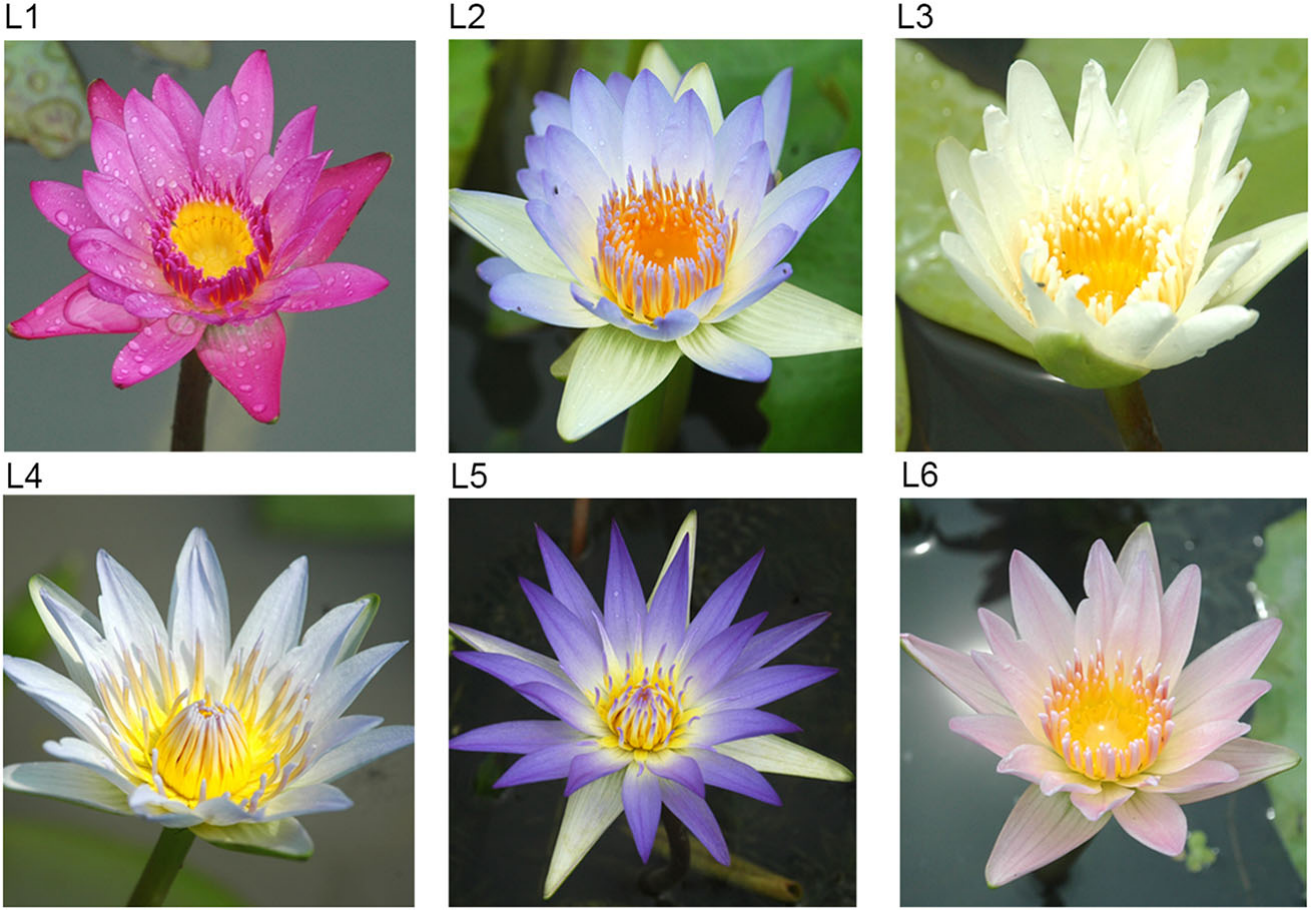
Flowers of the tropical water lilies that can overwinter in Hangzhou. L1–L6 lines showed the transformed plants

In the spring of 2015, ~1 million seeds were selected for germination in water with 10 mg/L HygB. A total of 2605 seedlings were obtained after transplanting, in which 2013 seedlings survived and were planted into the field. The winter temperatures in the testing field (in Hangzhou, China) from the end of 2015 to the beginning of 2016 fell to the lowest during the past 30 years, with a minimum temperature of −8.9 °C (Fig. 3a). Tropical water lilies could successfully overwinter in the humid tropical city of Xiamen, China, because this area never froze and seldom dropped to below 4 °C (Fig. 3b). However, the water surface was frozen successively of days in the city of Hangzhou, China (Fig. 3b), with the thickest ice layer reaching 4 cm. The ice did not melt on all days in January 23–24, 2016 (Fig. 3c). Through the whole winter experiment, we have obtained six putatively transformed lines that could overwinter outside in Hangzhou, China, and they bloomed well as shown in Fig. 4 during the winter season in Hangzhou. The surviving rate of cold stress-tolerant plants was 6/2013 = 2.98‰.

In February 15, 2016, the whole field of the water surface was frozen, all the non-transgenic control plants died (Fig. 3d), while the putatively transgenic plants had two fresh leaves frozen in the ice (Fig. 3e). On May 5, 2016, the floating leaves of the putatively transformed plants grew well with 20 pieces of floating leaves. The largest floating leaves had a diameter of 20 cm (Fig. 3f). On May 18, 2016, the first flower of the putatively transformed plants opened. Taken together, the highest cultivation latitude for the tropical water lily expanded from 24.3°N to 30.3°N, which increased the planting range by six latitudes (Fig. 3g).

In Hangzhou, these transgenic plants emerged in the open field in March, developed considerable green leaves in April, and flowered in late May (Fig. 4). The non-transgenic tropical water lily was transplanted from the greenhouse to the open field in middle June in Hangzhou, and it flowered in July. The non-transgenic plants grew rapidly with the rising of the water temperature, and grow best in summer.

### Transgene detection

**Fig. 5:**
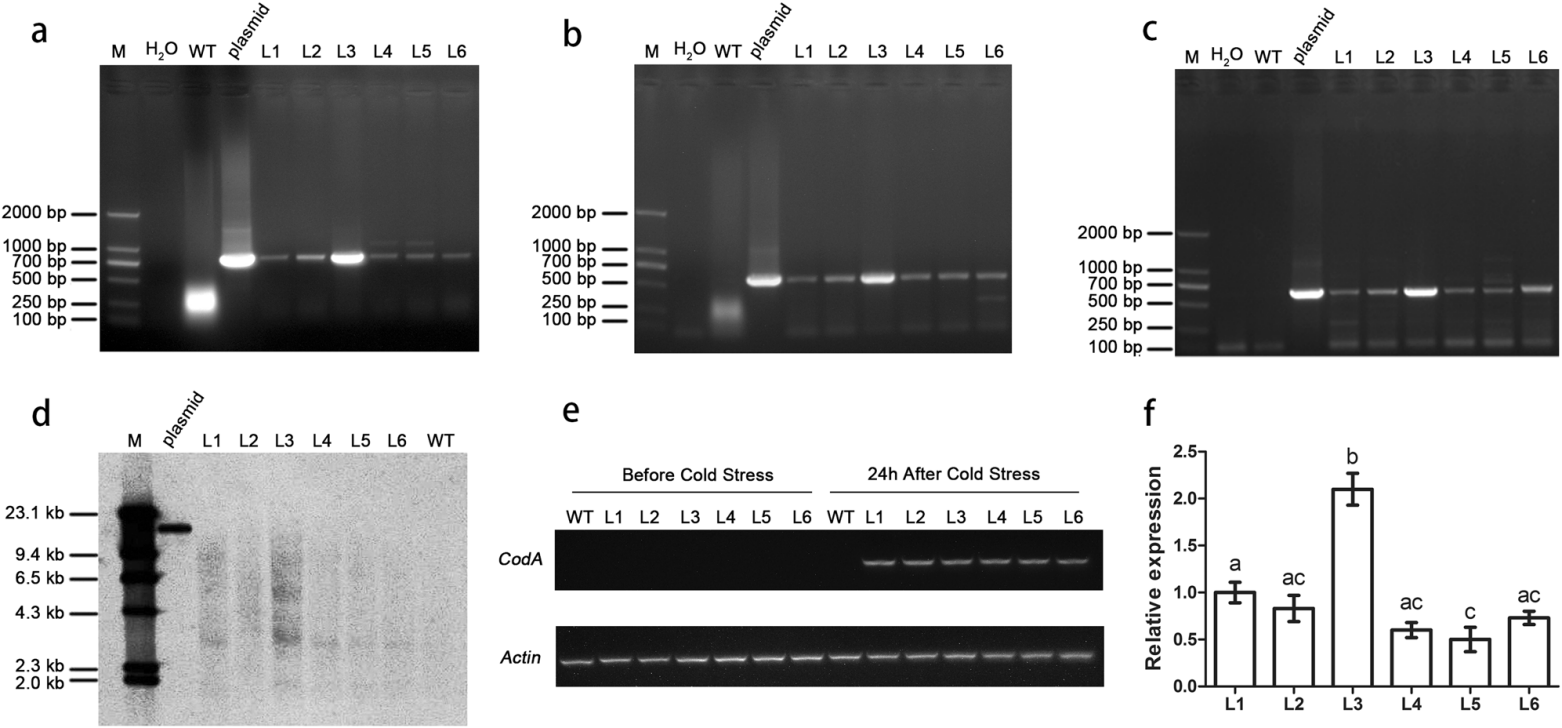
Transgene detection and expression of all the six lines. **a** PCR detection of *Rd29A* promoter (M DNA marker, H_2_O ddH_2_O, WT wild type, plasmid pBS1305RdcodA, L1–L6 transformed plants). **b** PCR detection of *Hyg* gene (M DNA marker, H_2_O ddH_2_O, WT wild type, plasmid pBS1305RdcodA, L1–L6 transformed plants). **c** PCR detection of *CodA* gene (M DNA marker, H_2_O ddH_2_O, WT wild type, plasmid pBS1305RdcodA, L1–L6 transformed plants). **d** The southern blot result presented the integration of *CodA* gene into the water lily genome. **e** RT-PCR analysis of *CodA* expression under cold treatment (WT wild type, L1–L6 transformed plants). **f** qRT-PCR analysis of *CodA* expression under cold treatment. L1–L6 are transformed plants. Using averaged expression of three samples of L1 as a unit, the relative averaged expression of the other five lines was compared to L1. Different letters indicate a significant difference (more than ±2.0-fold) between the six lines

Six lines of a transgenic water lily that could overwinter were obtained in this test, and were then detected by PCR amplification. The *Rd29A* promoter, *Hyg* gene, and the *CodA* gene were detected and amplified from all six lines (Fig. 5a–c). Meanwhile, among the non-transgenic plants none of the three products were detected. To confirm the integration of exogenous gene into the water lily genome and to reveal its copy number, six independent lines were selected for southern blotting. The results (Fig. 5d) showed that the *CodA* gene had been stably integrated into all the six transgenic lines; the dot color indicates that approximately two copies were inserted in L3, and only one copy was inserted in the other lines. The transformation efficiency (see Method) was calculated to have reached 1%.

The cold-induced expression profile of *CodA* was examined in the following scenarios: leaves of two transgenic lines, the non-transgenic plant grown under normal conditions, and the plants exposed to cold stress at 4 °C for 24 h. The *CodA* gene was driven by the stress-inducible *Rd29A* promoter, which is activated by abiotic stresses such as salt and cold^30^. No expression of *CodA* gene was observed in the control plants either before or after cold stress. Indeed, elevated expression of *CodA* was detected by RT-PCR in all the transgenic lines after cold stress (Fig. 5e). Furthermore, to compare the performance among the six lines at the 24-h cold stress point, qRT-PCR detection of the six lines suggests that line 3 (L3) has a significant higher expression than the other lines, whereas L5 had a significant lower expression than the L1 and L3 lines (Fig. 5f). These results indicate that *CodA* in the transformed plants had been successfully induced to express cold stress using the *Rd29A* promoter in the transformed tropical water lily. Taken together, these results showed that the *CodA* gene had been stably integrated into all of the six lines.

### Morphological and physiological performance under cold stress

**Fig. 6:**
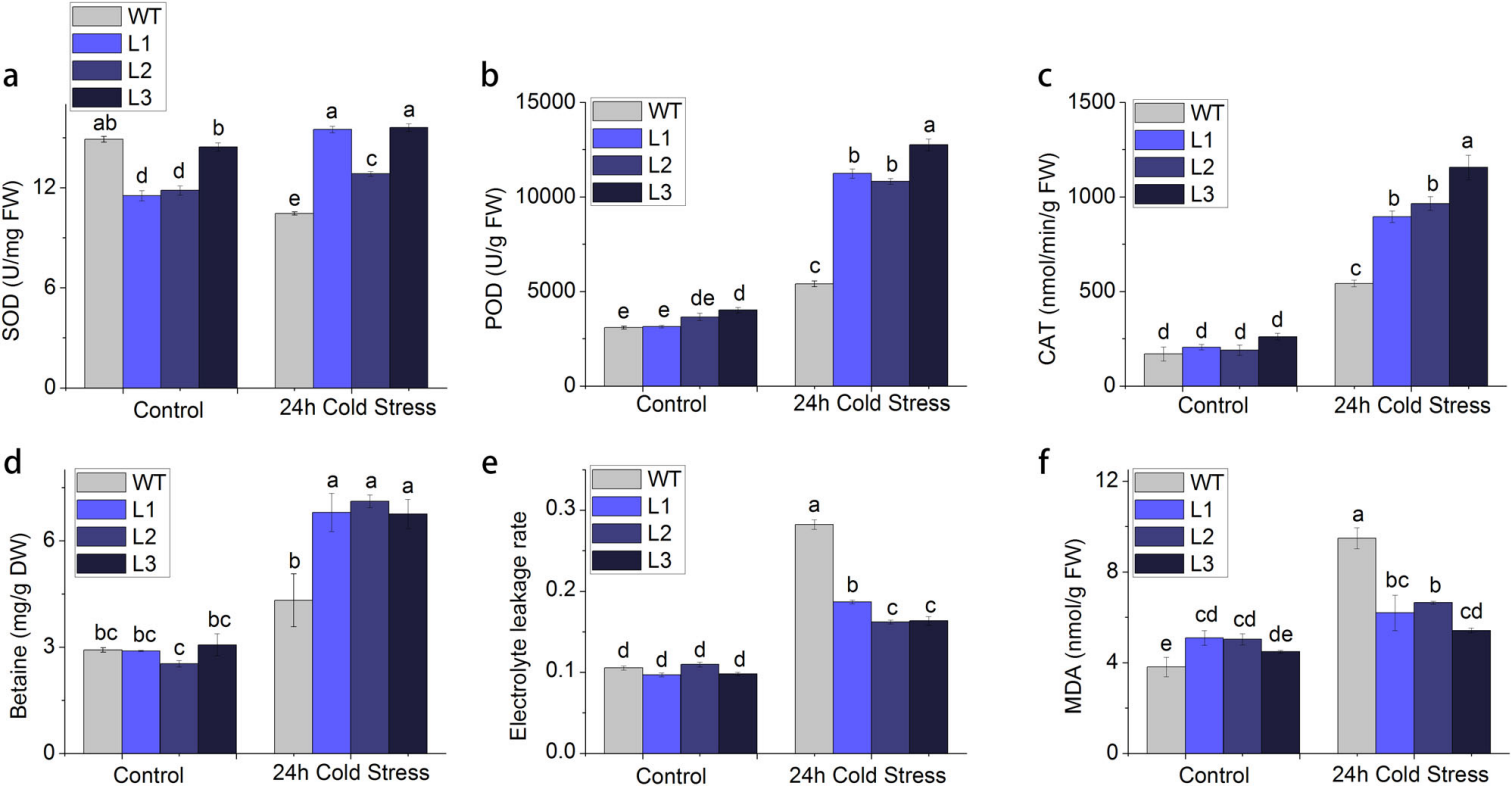
Comparison of morphological and physiological performance under cold stress. Different letters indicate significant differences for content comparisons (*α* = 0.05). **a** SOD activity, **b** POD activity, **c** CAT activity, **d** betaine content, **e** conductivity, and **f** MDA content

After 24 h of low-temperature (4 °C) stress, non-transgenic plant leaves were significantly more damaged than the transgenic plants. After 2 weeks, the leaves of non-transgenic plants damaged more severely. Although the leaves of transgenic plants were damaged, the terminal buds developed well, and the new leaves flourished. According to their best cold stress performance in Fig. 5f, we selected L1, L2, and L3 for physiological investigation; we found that the activity of SOD (Fig. 6a), POD (Fig. 6b), and CAT (Fig. 6c); and the increased content of betaine (Fig. 6d) were higher in the transgenic plants than in the non-transgenic plants after cold stress treatment. In contrast, both the conductivity (Fig. 6e) and MDA content (Fig. 6f) were lower in the transgenic plants than in the non-transgenic plants. These results suggest that the expression of exogenous *CodA* gene could trigger physiological responses via increasing the betaine content and thus improving the cold stress tolerance of transgenic plants.

## Discussion

Worldwide water lilies and lotuses are the top two aquatic ornamental plants. The breeding of water lilies is developing rapidly in Thailand, the United States, and China. The International Water Lily and Water Gardening Society is authorized to register new varieties of water lilies around the world^14^. All the registered varieties so far are natural mutants or hybrids by crossbreeding. Although traditional crossbreeding of water lilies yielded hundreds of novel cultivars, it is often time-consuming, labor-intensive, and very expensive. Breeding a new species of water lily requires a large germplasm nursery, reliance on extensive screening, hard work in the field, and low probability of luck, waiting and observation for more than 2 years. People need to hybridize the water lily in June and harvest the seeds in July. Then, sow the seeds and they will flower in the September of October. Select the best-performed lines and see whether the phenotype is stable. The next year, if the phenotype is stable, one can register the water lily in the International Water Lily Organization. Our molecular breeding of water lily, including the genetic transformation through pollen-tube pathway, large-scale screening of transformed seeds (thousands of seeds from ~1 million seeds), and physiological tests and molecular detection provides a novel protocol for water lily breeding. In breeding the transgenic water lily, in the first year, breeders needed to prepare the plasmid carrying the transgene, and culture the agrobacteria. It will take about 2 months of lab work. In June, infect the flower in the field at noon. The harvested seeds need to be screened with Hyg. Then, the seedlings will be tested in the field, such as cold stress test in this study. In the lab, the potential transformed plants need a series of molecular tests for the transgene, such as PCR, qRT-PCR, southern blot, transgene copy detection, physiological changes, and so on. These experiments together will take about half-year. The methods and reagents for the water lily are always a bit different for the model plant *Arabidopsis*. So these experiments on a specific water lily will require a graduate or a master student, guided by a skilled teacher with molecular biology background. Another important advantage of a transgenic breeding approach over the traditional approach is its introduction of untraditional traits, such as the introduction of cold stress-tolerant trait into a tropical water lily, and perhaps in the future, we are able to edit any trait we wish through gene editing technology. Therefore, our work will hopefully promote the development of the commercial water lily varieties and the related industries such as cut flowers, vegetables, and essential oils. Our work serves as the first study using molecular technology in breeding water lilies, which is a milestone in water lily breeding industry.

Although we have obtained six lines of transgenic water lilies with beautiful blooms, the water lilies used in this study were genetically unstable hybrids with little economic value, which we think were just intermediate materials in the whole breeding program, and the whole transgenic pipeline was just an intermediate step in the whole breeding program. Based on our established pipeline, in the future, we will continue to carry out and test the following two methods: (i) hybrid the transgenic water lilies with non-transgenic ones and (ii) transform the *CodA* gene into other target water lilies. Based on these excellent water lilies, we will carry out the transgene zygosity experiment. However, it should be noted that to obtain these genetic stable transgenic water lilies, it will take another 3–5 years.

In this study, the transgenic tropical water lilies in Hangzhou that overwintered outside still possessed the excellent traits of the parent tropical water lilies. Therefore, the highest cultivation latitude for a tropical water lily has expanded from 24.3°N to 30.3°N. The latitude limit for planting water lilies was advanced 6° northward. In the future, we will set up a series of latitudes for overwinter tests. The test points have been scheduled to locate in Hancheng (35.5°N latitude), Beijing (39.3°N latitude), and Harbin (45°N latitude). We will also use the transformation system of the tropical water lily to test the performance of other functional genes in water lilies. The modified tropical water lilies are great breeding parents to introduce the new cold stress-tolerant character to those tropical water lilies with different floral features, flower colors, and leaf colors. Since water lilies have two reproductive strategies, asexual reproduction, and sexual reproduction^43^, the introduced novel traits would be easily maintained. Although the current genetic transformation efficiency of water lilies based on the pollen-tube channel method reached only 1‰, the number of seeds of a single flower of a tropical water lily can amount up to 10,000 and even more so, the transformation efficiency can be greatly compensated compared to other crop plants. Thus, the water lily genetic transformation efficiency is not that low at all. At present, there is no established aquatic tissue culture system for the water lilies or lotuses. In the future, we will strive to establish a water lily tissue culture-based transgenic pipeline to vigorously promote molecular breeding of water lilies.

In addition to its values in breeding, the transgenic system is valuable for functional studies of genes in the postgenomic era of the water lily research. Water lilies are potential model plants for basal angiosperms^44^, attracting many plant biologists to sequence its genome. With the completion of genome sequencing of the tropical *Nymphaea colorata*^13^, functional studies of genes require transgenic systems to validate the functions such as genes related to floral color, secondary metabolism, stress, and immune signaling^45^. This study shows great potential that the evo-devo studies of basal angiosperms are entering a new era.

## Electronic supplementary material

Supplementary Tables
