# Supplementary material for: Molecular breeding of water lily: engineering cold stress tolerance into tropical water lily
Source: Hortic Res. 2018 Nov 30;5:73. doi: 10.1038/s41438-018-0086-2 (PMC6265338; doi:10.1038/s41438-018-0086-2)
Supplement: Supplementary file 1 — Supplementary Tables [file 41438_2018_86_MOESM1_ESM.docx]

**Supplementary Tables**

**Table S1.** Effects of Agrobacterium concentration on transformation rate and seed setting rate.

| Concentration | Transformation rate (TR) | Setting rate (SR) | TR*SR |
| --- | --- | --- | --- |
| 0 | 0 | 1 | 0 |
| OD600=0.47 | 0.03% | 95% | 0.000285 |
| OD600=0.7 | 0.10% | 80% | 0.0008 |
| OD600=0.93 | 0.14% | 42% | 0.000588 |
| OD600=1.05 | 0.16% | 13% | 0.000208 |

**Table S2.** Effects of Hygromycin B concentration on seedling rate.

| Concentration (mg·L^-1^) | Total seeds | seedlings | Emergence rate |
| --- | --- | --- | --- |
| 0 | 3000 | 1890 | 63% |
| 5 | 3000 | 100 | 3.3% |
| 10 | 3000 | 10 | 0.3% |
| 15 | 3000 | 6 | 0.27% |
| 20 | 3000 | 2 | 0.07% |
| 25 | 3000 | 2 | 0.07% |
